# Neural and Behavioral Predictors of Treatment Efficacy on Mood Symptoms and Cognition in Mood Disorders: A Systematic Review

**DOI:** 10.3389/fpsyt.2018.00337

**Published:** 2018-07-26

**Authors:** Ida Seeberg, Hanne L. Kjaerstad, Kamilla W. Miskowiak

**Affiliations:** ^1^Neurocognition and Emotion in Affective Disorders Group, Copenhagen Affective Disorder Research Centre, Psychiatric Centre Copenhagen, Rigshospitalet, Copenhagen University Hospital, Copenhagen, Denmark; ^2^Department of Psychology, University of Copenhagen, Copenhagen, Denmark

**Keywords:** mood disorders, treatment, biomarker, cognition, neuroimaging, treatment efficacy, personalized medicine, precision medicine

## Abstract

**Background:** The clinical and etiological heterogeneity of mood disorders impede identification of effective treatments for the individual patient. This highlights a need for early neuronal and behavioral biomarkers for treatment efficacy, which can provide a basis for more personalized treatments. The present systematic review aimed to identify the most consistent neuronal and behavioral predictors of treatment efficacy on mood symptoms and cognitive impairment in mood disorders.

**Methods:** We identified and included 60 original peer-reviewed studies investigating neuroimaging and behavioral predictors of treatment efficacy within the domains of emotional and non-emotional cognition, structural neuroimaging, and resting state functional connectivity in patients with unipolar or bipolar disorder.

**Results:** Lower baseline responsivity in limbic regions coupled with heightened medial and dorsal prefrontal responses to emotional stimuli were the most consistent predictors of response to pharmacotherapy for depression. In contrast, heightened limbic and ventral prefrontal reactivity to emotional stimuli seemed to predict efficacy of psychological interventions. Early modulation of fronto-limbic activity and reduction in negative bias were also associated with treatment response. Better performance on non-emotional tests at baseline was relatively consistently associated with efficacy on mood symptoms, whereas the association between neural activity during non-emotional tests and treatment response was less clear. Other baseline factors associated with treatment response were greater white matter integrity, resting state functional connectivity, more prefrontal gray matter volume as well as an early increase following short administered treatment. Finally, emerging evidence indicates that baseline cognitive deficits are associated with greater chances of achieving treatment efficacy on cognition.

**Conclusions:** Patients' profile of emotional and non-emotional cognition and neural activity—and the early treatment-associated changes in neural and cognitive function—may be useful for guiding treatments for depression. While cognitive deficits at baseline seem to improve chances of treatment efficacy on cognition, more studies of this association are urgently needed.

## Introduction

Unipolar depression (UD) and bipolar disorder (BD) are among top contributors to the global burden of disease, with UD being the single largest contributor to global disability worldwide (1). At a global level, more than 300 million people suffer from UD and 60 million from BD (1) and the economic cost of these affective disorders is estimated to be €113 billion in Europe and $128 billion in the United States per year (2, 3). Despite the major economic imperatives to optimize treatment for these disorders, current treatment options are limited by insufficient efficacy on depressive symptoms for 30–40% of patients, general time lag of several months before an effective treatment can be identified for the individual patient, and frequent residual cognitive impairments (4–6). Specifically, cognitive impairment has emerged as a new treatment target based on evidence for persistent mild to moderate cognitive impairments beyond the acute illness episodes in both UD and BD that impede patients' functional recovery and quality of life (7, 8).

Presently, first-line treatments for mood disorders include pharmacological treatment with selective serotonin reuptake inhibitors (SSRI) and/or evidence-based psychotherapy, such as cognitive-behavioral therapy (CBT) (9). However, it is often unclear which treatment is optimal for the individual patient until after several weeks of treatment given the delay in the onset of response to pharmacotherapy and psychotherapy of 4–6 weeks (10). Therefore present clinical practice and guidelines for best treatment strategy relies on a trial and error approach and a pragmatic advice on switching to a different treatment after 4–6 weeks of continued treatment with a therapeutic dose. As most patients do not respond to their first prescribed treatment, this leads to a substantial time lag of several months before an effective treatment can be identified for the individual patient. In this time, patients' ability to work and function in everyday life is severely affected, resulting in great personal and socioeconomic costs. Notwithstanding, there is no clinically useful guideline as to which specific treatment would be the most optimal for the individual patient based on their particular symptom presentation (9–11).

The clinical and etiological heterogeneity of patients with mood disorders implicates distinct pathophysiological profiles for each individual patient (12, 13). This highlights a pressing need for identification of biomarkers of treatment efficacy that can serve as a platform for personalized treatments. A major focus of recent research has therefore been to identify associations between biological or psychological measures and treatment response. Clinical predictors of treatment efficacy, such as severity or depressive clinical subtypes, have so far shown disappointing predictive value of improvement on mood symptoms. Genetic testing, neuroimaging, psychological and psychophysiological approaches have therefore been employed (9, 14). In particular, identification of early predictors of antidepressant response at neural and cognitive level seems an essential step to more effectively personalized treatment options (10). However, the findings from the large number of neuroimaging and behavioral studies of efficacy markers vary, and there is no clear understanding of what are the most robust neurocognitive biomarkers of treatment efficacy on mood symptoms.

More recently, studies have begun to investigate treatments that directly target residual cognitive impairments in mood disorders. Specifically, recent studies have found global or selective cognitive difficulties in 50–70% remitted patients with mood disorders, and poorer quality of life, more stress and impaired work capacity in cognitively impaired patients relatively to those who are “cognitively intact” (8). While there is currently no clinically available treatment for cognitive impairment in mood disorders, there is strong preliminary evidence for several candidate treatments including, modafinil, vortioxetine, erythropoietin, lurasidone and cognitive remediation (15–20). Nevertheless, treatment development targeting cognition is hampered by the lack of insight into the neurobiological underpinnings of cognitive improvement and lack insight into baseline predictors of efficacy on cognition.

Taken together, there is a need for insight into what are the most robust biomarkers of treatment efficacy on mood symptoms and cognition to aid more effective, personalized treatments. The aim of the present systematic review is therefore to provide a “landscape” view of putative functional and structural neuroimaging and behavioral predictors of treatment efficacy on depressive symptoms and cognition in mood disorders. Based on this, a discussion will be formed on the identified putative biomarkers and how they may be integrated in the clinical assessments of patients to aid speed and efficacy of future treatment strategies.

## Methods

### Selection criteria

The initial search criteria were defined in accordance to PICO framework (Population, Intervention, Comparison, Outcome). We included original peer-reviewed articles involving predictive intervention studies on patients with a mood disorder (unipolar or bipolar disorder) and no comorbid schizophrenia/schizoaffective disorder. The primary criterion was a focus on prediction of treatment response or remission using either neuroimaging (fMRI or MRI) or objective neurocognitive testing as a measure. We excluded articles that were naturalistic in design with no control of treatment; involved pediatric, adolescent or geriatric participants; only assessed cognitive measures with subjectively informed ratings; only looked at single drug administrations (with no follow-up of treatment efficacy following longer term treatment). Other reasons for exclusion were: other languages than English; meeting abstract, case report or study protocol; animal studies; comorbid schizoaffective disorder.

### Search strategy

A systematic computerized search was performed on PubMed/MEDLINE, EMBASE and PsychInfo databases from inception up until October 2017. The search profile included three elements: “biomarker,” “neuroimaging OR cognition” and “mood disorder AND therapy/treatment” with each their combinations in the respective databases (see supplementary material for more details).

**Figure 1:**
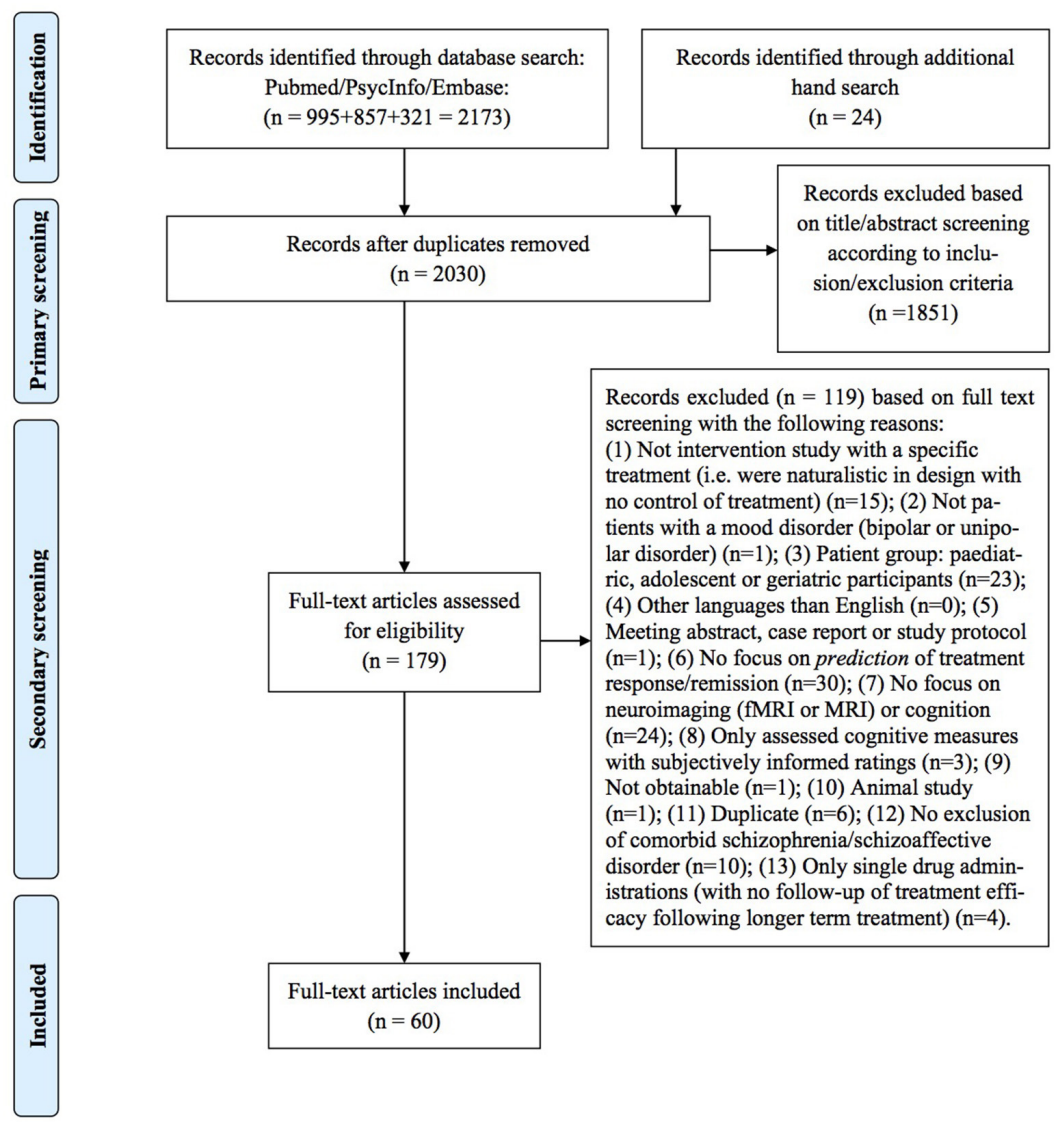
PRISMA flow-chart.

Two of the authors (IS and HLK) independently performed a primary title/abstract screening for potentially eligible articles and, following this, a secondary full-text screening was conducted. In both primary and secondary screening, we considered each of the unique references according to inclusion/exclusion criteria, but only provided information on the specific reasons for exclusion of papers in the secondary screening. Finally, a hand search was performed by tracking and screening the citations of the included articles for possible extra inclusions. Agreement between the two authors who performed the screening (IS and HK) was good (primary: 94%, secondary: 87%), and any disagreements were discussed and consensus was reached in all cases. This systematic review has followed the procedures of the *Preferred reporting items for systematic reviews and meta-analysis* (PRISMA) statement (21), and a PRISMA flowchart can be seen in Figure 1.

## Results

The initial screening identified 2030 articles (duplicate hits excluded). Out of these, 158 articles were included for titles/abstracts screening, resulting in 89 full-text articles evaluated for eligibility. Of these, 60 met inclusion criteria and were included in this review. In total, 58 studies explored different predictors of treatment efficacy on *mood symptoms*: 26 investigated “hot” (emotional) cognition with behavioral and functional neuroimaging measures, 15 investigated neuroimaging and behavioral measures of “cold” (non-emotional) cognition as predictors of treatment response on mood symptoms, ten investigated resting-state functional neuroimaging measures, and eight investigated structural neuroimaging measures. Only two studies investigated predictors of treatment efficacy on *cognition* using demographic variables and behavioral measures of cognition.

### Emotional cognition to predict treatment response on mood symptoms

The 26 studies of “hot” cognition as a putative biomarker of treatment response examined behavioral and neuroimaging measures of facial emotion processing and regulation, implicit processing of emotion pictures, emotional self-referential processing, and reward processing (see Supplementary Table 1 for study details).

#### Behavioral measures of emotion processing

Only one published study to date examined early change in behavioral assays of emotion processing as a predictor of subsequent treatment response (22). The study revealed that early increase in the recognition of happy facial expressions in the first 2 weeks of treatment with the SSRI citalopram or the SNRI reboxetine predicted clinical improvement indicated by reduction in total score on Clinical Outcome in Routine Evaluation (CORE). In contrast, early treatment-associated improvements in the recognition of disgust and surprise were not associated with treatment response.

#### Neuroimaging data on emotion processing

##### Neural response to emotional faces

Fourteen studies explored whether treatment response was associated with baseline neural activity to emotional faces or with the early effects of treatment on neural response to emotional faces in UD or BD (23–36).

Overall, the studies found that those patients who went on to respond to antidepressant treatment (i.e., treatment responders) exhibited amygdala *hypo-*activity to negative facial expressions at baseline compared to healthy controls (32, 34). Further, one study compared responders to healthy controls and specified that the association between baseline amygdala *hypo-*activity to facial emotions signaling reward and threat and treatment response was independent of medication type (SSRIs escitalopram or sertraline vs. SNRI venlafaxine). In keeping with this, the study reported an association between amygdala *hyper-*activity to sad facial expression and non-response to venlafaxine compared to healthy controls (32). One study found that this association between amygdala *hypo-*activity to sad faces and subsequent treatment response to the anticholinergic antidepressant scopalomine could only be observed when patients were required to attend to the faces (i.e., instructed to focus explicitly on the faces' portion of the picture) in the presence of meaningful distractor stimuli (34). Consistent with this, one study reported that lowered baseline activity in middle occipital cortex (i.e., visual processing area) during encoding and recognition of faces with (task-irrelevant) emotional expressions was associated with better treatment response to scopalomine (29). Notably, Redlich et al. (36) did not find any significant predictors of treatment response to electroconvulsive therapy (ECT), though reported that both treatment groups (ECT and pharmacotherapy) showed increased amygdala reactivity to sad faces at baseline compared to healthy controls.

Studies have also found consistent associations between responses to either drug treatment or chronotherapy (combinations of repeated total sleep deprivation and light therapy) and baseline prefrontal activity in UD and BD (23, 27, 31). Specifically, UD responders to SSRIs fluoxetine or sertraline exhibited *greater* anterior cingulate cortex (ACC) response to fearful, angry and sad faces compared to non-responders (23, 30, 31). In keeping with this, BD-I responders to chronotherapy showed greater ACC and medial prefrontal cortex (MPFC) response during implicit processing of fearful and angry facial expressions compared to non-responders (31). Further, greater dorsomedial PFC (DMPFC) and posterior cingulate cortex (PCC) response to sad faces in UD was associated with better response to treatment with the noradrenergic and specific serotonergic antidepressant (NaSSA) mirtazapine or venlafaxine (27).

Finally, three studies reported an association between greater functional connectivity (FC) (i.e., closer to healthy controls) and treatment response to chronotherapy or pharmacotherapy (26, 31, 35). In particular, two studies found that responders to either chronotherapy or SSRI showed greater baseline activity and FC within fronto-limbic networks during implicit processing of fearful and angry faces (31, 35). In accordance with this, responders to either venlafaxine or mirtazapine had increased FC of the orbitofrontal cortex (OFC) in the left precentral gyrus and internally within the right middle OFC during an emotional face-matching task at baseline (26).

Notably, two studies of CBT revealed the opposite association between baseline neural activity and treatment response (24, 25). Specifically, responders to CBT displayed *lower* dorsal ACC (DACC) activity to sad faces at baseline compared to non-responders (24). In fact, responders' DACC response to sad faces was more “normal” (i.e., more similar to the activity in healthy controls) than non-responders'. Moreover, one study reported that the FC during processing of sad faces at the lowest and highest intensities identified patients who had a full clinical response to CBT (25).

Only a few studies (all conducted on patients with UD) explored whether *early changes* in neural response to emotional faces can predict subsequent treatment efficacy (28, 29, 33). Nevertheless, these studies provide consistent evidence for early modulation of limbic-subcortical-prefrontal brain networks being predictive of subsequent treatment efficacy. One study found that early treatment-related *decrease* (toward normal levels) in ACC, insula, amygdala, and thalamus reactivity to fearful faces after 1 week characterized responders vs. non-responders to escitalopram (33). In contrast, responders to the SSRI paroxetine showed an early treatment-related *decrease* in amygdala response to negative faces (relative to baseline) compared to non-responders, as well as an *increase* in lower dorsal regions (DLPFC and DMPFC) to negative faces (toward “normal” levels) after six weeks of treatment relative to non-responders (28). This suggests that paroxetine exposure over time improves dorsal prefrontal regulation of abnormal limbic activity. Another study found that following short-term (1 week) scopalomine administration, responders exhibited *increased* response (closer to “normal”) in bilateral middle occipital cortex (relative to baseline) during encoding and recognition of faces with (task-irrelevant) emotional expressions (29).

##### Neural response to emotional pictures

Five fMRI studies investigated the predictive value of baseline neural activity during emotional reactivity and emotional regulation using emotion-laden picture stimuli for efficacy of pharmacotherapy in UD (37–41). Responders to a combination treatment with fluoxetine and antipsychotic olanzapine or to venlafaxine were characterized by *greater* baseline activity to *negative* images in the ACC and premotor cortex and to *positive* images in the posterior cingulate gyrus and precuneus than non-responders (37, 40). Further, *lower* ventrolateral PFC (VLPFC) activity at baseline during attempts to suppress positive emotions elicited by pleasant pictures was associated with greater reduction in anhedonia after venlafaxine or fluoxetine treatment (38). Finally, a study of CBT found that *greater* pre-treatment activity in the DLPFC and anterior temporal lobe (ATL) to negative images and to pictures in general (emotional and neutral) predicted symptom improvement after CBT (39).

Only one study investigated the predictive value of early changes in neural activity in response to the mood-stabilizer lithium or the atypical antipsychotic quetiapine for subsequent treatment efficacy (41). Patients with BD-I who later achieved remission exhibited an early *increase* in MPFC, temporal, and posterior cortical areas to emotional (unpleasant) pictures after 1 week of either lithium or quetiapine treatment. Also, while amygdala activity at baseline and after short-term treatment administration did not predict treatment remission, non-remission was associated with baseline *hypo*-activity in the amygdala in comparison to healthy controls and remitted subjects (41).

##### Neural response during emotional self-referential processing

Four fMRI studies explored the predictive value of neural response during emotional self-referential processing at baseline (i.e., in which patients were instructed to judge the personal relevance of a picture or word) (42–45). Delaveau et al. (45) found that UD remitters to treatment with the atypical antidepressant agomelatine (melatonin and serotonin receptor antagonist) displayed lower baseline activation in the rostral DMPFC, PCC, and DLPFC during self-referential processing of emotional and neutral pictures compared to non-remitters. Consistent with this, Miller et al. (44) found that UD responders to escitalopram displayed lower baseline responses to negative self-referent words in midbrain, DLPFC, paracingulate, ACC, thalamus and caudate nuclei compared to non-responders.

Two studies of baseline neural activity predictors for response to CBT revealed a somewhat different pattern (42, 43). Specifically, patients with amygdala *hyper*-activity and low subgenual ACC (sgACC) reactivity during self-referent processing of negative emotional words displayed the most improvement after CBT (42, 43). In addition sgACC activity remained low for patients in remission after treatment, suggesting that successful treatment did not operate by normalizing this mechanism but rather by remaining more like healthy individuals from pretreatment to posttreatment measurements (43). Notably, no studies of self-referent processing explored the association between early neural activity changes and subsequent clinical improvement in response to pharmacological or psychological interventions.

##### Neuroimaging and behavioral measures of reward processing

Two studies of reward processing as a putative biomarker for response to behavioral activation therapy for depression (BATD) (46, 47) found that neural response to monetary awards at baseline was predictive of treatment efficacy. Specifically, increased frontostriatal connectivity during reward anticipation and increased capacity to sustain ACC activity when receiving rewards, were found to be associated with greater treatment response (46, 47). Consistent with this, Carl et al. (46) reported that greater change in reaction time during reward trials (i.e., faster response at run 2) at baseline predicted treatment response.

#### Interim summary

In sum, responders to pharmacotherapy tend to be characterized by heightened PFC top-down control as well as *greater* recruitment of ACC and/or lowered limbic and visual cortical reactivity during the processing of emotional stimuli than non-responders. Accordingly, particularly *less* limbic and occipital reactivity to task-irrelevant emotional aspects of faces in preference for cognitively demanding tasks was associated with better antidepressant efficacy. In contrast, responders to psychological interventions seem to be characterized by a pattern of *greater* baseline limbic reactivity, *less* PFC response to emotional stimuli and *greater* neural response to reward stimuli.

Additionally, the small number of studies investigating *change* in neural activity after 1–6 weeks of pharmacological treatment consistently indicate that treatment-related reduction in limbic activity and increase in DPFC top-down control to negative valence emotional stimuli are putative early predictors of response to distinct biological interventions.

### Non-emotional cognition to predict treatment response on mood symptoms

A total of 13 studies investigated “cold” cognition as a putative biomarker for treatment response on mood symptoms (see Supplementary Table 2 for study details). Five studies assessed neuroimaging measures, while eight studies examined purely behavioral assays of non-emotional cognition as biomarkers of treatment efficacy (7, 15, 48–58).

#### Combined neuroimaging and behavioral measures of non-emotional cognition

Five studies used a combination of neuroimaging and neurocognitive assessment to investigate baseline predictors for treatment response on mood symptoms in patients with UD (51, 52, 55, 57, 58). Two studies of inhibitory control found that aberrant neural activity within prefrontal regions during a parametric Go/No-go task predicted subsequent treatment efficacy. In one study, patients who responded well to duloxetine, escitalopram or citalopram treatment showed more *unsuccessful* inhibition (i.e., more performance errors) at baseline as well as less recruitment (i.e., *lower activation)* of brain areas important for cognitive control and/or interference resolution, including the ACC, left VMPFC and right VLPFC (58). In contrast, the other study found that *greater* rostral ACC activation during *unsuccessful* inhibition was associated with subsequent treatment response (51).

Two studies of patients with UD found an association between aberrant neural activity during verbal working memory and clinical response to fluoxetine and rTMS, respectively (52, 57). Specifically, the fluoxetine study reported *lower* DACC during verbal n-back working memory at baseline in responders vs. non-responders (52). In contrast, the rTMS study found that responders displayed a *combination* of lower activity in perigenual, medial OFC, and middle frontal cortices, and a greater activation in the ventral-caudal putamen during a word-generation task at baseline compared to non-responders (57). Notably, a third study (55) found no significant associations between treatment response to rTMS and structural or behavioral measures at baseline.

#### Behavioral measures of non-emotional cognition

Seven studies explored purely *behavioral* assays of non-emotional baseline cognition (i.e., performance accuracy and speed) as potential predictors of treatment response on depression symptoms in patients with UD and BD (7, 15, 48–50, 53, 54).

Four studies reported that *better* cognitive performance across several domains predicted response to antidepressant treatment (7, 48, 50, 53). In particular, two studies of UD patients found that responders to fluoxetine displayed better baseline performance in executive functioning and mental processing speed than non-responders (48, 50). The third study showed that greater attention performance at baseline was associated with better clinical outcome in UD patients treated with agomelatine (7). Finally, the fourth study found that UD and BD patients with less over-general memory (i.e., lack of memory for specific episodes, which is a key feature of depression) recovered faster from depression and were at lower risk for relapse after ECT (53).

In contrast, one study found that *poor* cognitive performance predicted subsequent clinical response. Specifically, UD responders to the new norepinephrine–dopamine reuptake inhibitor bupropion displayed visual memory deficits and slowed mental processing speed at baseline (15).

Finally, two studies showed that a *combination* of high and low performance within different cognitive domains predicted treatment response to SSRIs (49, 54). In particular, one study found that a combination of good sustained attention performance and poor psychomotor speed and planning performance predicted response to fluoxetine (54). A similar pattern of good cognitive performance on “simple” tasks and poor performance in “complex” tasks (requiring more demanding effort and maintenance) was observed in responders vs. non-responders to SSRI (drug not specified) (49).

Only one study investigated *early change* in cognition as a biomarker for treatment response in patients with UD and BD (56). This study revealed that early improvement in visuospatial memory after 2–3 weeks of rTMS treatment predicted eventual treatment response. Interestingly, the initial cognitive changes appeared to be independent of antidepressant efficacy, since patients' cognitive improvement occurred *before* any mood changes. In contrast, early treatment-related improvements in verbal learning and memory and attention span were not related to subsequent treatment response (56).

#### Interim summary

Taken together, findings from functional neuroimaging studies highlight aberrant neural activity during non-emotional cognition tasks as a putative prognostic biomarker. The most consistent marker of treatment response seems to be less recruitment of dorsal PFC during cognitive performance. Studies of *behavioral* performance measures on non-emotional cognitive tests in UD and BD patients provided more consistent findings. Specifically, it was demonstrated in several studies that *high* performance across attention, executive function, and memory tests was a predictor of response to pharmacological interventions (SSRI and SNRI), rTMS, and ECT for depression (7, 48, 50, 53). Additionally, a couple of other studies found that a combination of good performance on simple tests and poor performance on complex tests might predict treatment efficacy (49, 54).

### Combined emotional and non-emotional cognition to predict mood improvement

Two studies investigated whether a pattern of emotional and non-emotional cognitive performance could be used to identify the individual patients who would achieve clinical remission in response to escitalopram, sertraline, or venlafaxine (11, 59) (see Supplementary Table 3 for study details). Using a novel pattern classification analysis, Etkin et al. (59) and colleagues showed poorer treatment outcomes for a subgroup of depressed participants (approximately one-quarter of patients) with impairment across most cognitive and emotional capacities. High task performance predicted remission after escitalopram treatment (but not other medications) with 72% accuracy. The other study investigated whether predictions of remission could be made with a larger cognitive assessment battery, including eight non-emotional tests tapping into several cognitive domains and one emotional identification test (11). The study reported that the test battery thresholds established a negative predictive value of ≥80%, which identified 41% of participants not remitting on escitalopram, 77% of participants not remitting on sertraline, and 39% of participants not remitting on venlafaxine (all including 20% false negatives). These findings provide promising ways to predict treatment efficacy with a high sensitivity and specificity for each individual patient, although replication is needed to establish an applicable tool for clinical use.

### Resting state functional connectivity to predict treatment response on mood symptoms

Ten studies investigated the association between resting state and FC at baseline and treatment response in UD and BD (9, 10, 60–67) (see Supplementary Table 4 for study details).

Four of these found that greater baseline resting-state FC in the PFC predicted response to treatment with rTMS applied to either DLPFC or DMPFC (60–62, 67). Specifically, Ge et al. (67) found that both UD and BD patients who responded well to treatment displayed enhanced contributions to functional connectivity, namely hyper-connectivity in ACC/VMPFC within the anterior default mode network (DMN) and DACC/insula within the salience network. Downar et al. (60) found that at baseline, UD and BD responders showed increased connectivity within reward pathways, including left VMPFC, the ventral tegmental area, and striatum compared with non-responders. Liston et al. (61) found that baseline hyperconnectivity between the sgACC and multiple areas of the DMN and central executive network independently predicted greater clinical improvements after TMS. Finally, Salomons et al. (62) found that higher baseline FC between DLPFC and a medial prefrontal cluster spanning the subgenual cingulate gyrus and DMPFC was associated with better response to treatment in UD and BD. In addition, patients with low baseline cortico-thalamic (DMPFC-medial dorsal thalamus), cortico-striatal (DMPFC-putamen), and cortico-limbic (sgACC-amygdala and sgACC-hippocampus) connectivity also experienced a greater response to the treatment (62).

Among UD patients treated with BATD, responders were characterized by greater baseline connectivity between the right insula and right middle temporal gyrus (63). For UD patients given CBT, remission was predicted by greater positive FC at baseline with the subcallosal cingulate cortex and three regions: the dorsal midbrain, VLPFC/insula, and VMPFC (9). The latter study also compared patients receiving CBT with those treated with antidepressant medications (escitalopram or duloxetine). This revealed that *negative* FC (i.e., anti-correlations of activity over time) between the same regions was associated with remission in medication-treated patients (9). The study thus suggests that the direction of baseline FC can be used to predict whether an individual is more likely to benefit from psychotherapy or pharmacotherapy. Finally, assessment of UD patients with treatment-resistant depression (TRD) receiving ECT (64) revealed that the brain areas in which resting-state activity provided the largest contribution in predicting subsequent remission were the cingulate cortex, medial- and orbitofrontal cortices, although the direction of connectivity (increased vs. decreased) was not specified.

Four studies of both UD and BD patients found evidence of *early change* in neural activity during resting state that predicted subsequent response to various biological treatments (10, 62, 65, 66). Specifically, efficacy of rTMS and ECT was associated with early *increase* in positive/negative fronto-limbic connectivity, respectively (62, 65). Also, another study using transcutaneous vagus nerve stimulation (tVNS) for treatment of UD found that treatment responders exhibited early increase in left anterior insula activity after the first treatment session (66). In contrast, Cheng et al. (10) found that responders to escitalopram were characterized by early decrease in occipital cortex activity and increase in DLPFC, DMPFC, and middle cingulate cortex activity only 5 h after initial treatment administration. However, the best predictor of clinical remission was increased activity in ACC, midcingulate cortex, and right superior temporal gyrus (STG) 5 h after administration of escitalopram to endpoint. Moreover, aberrant (increased and decreased, respectively) FC between cingulate and limbic areas was predictive of response in patients who were treated with either ECT or rTMS (62, 65).

#### Interim summary

Taken together, the findings support the idea that greater positive fronto-limbic connectivity during resting state at baseline is predictive of treatment response to a psychotherapeutic intervention. In contrast, decreased fronto-limbic connectivity is predictive of response for antidepressant medication treatment. Additionally, response to antidepressant treatment was associated with early *increase* (positive and negative) in fronto-limbic connectivity during resting state.

### Structural abnormalities to predict treatment response on mood symptoms

#### Grey matter

Four studies investigated the association between gray matter (GM) and treatment response in UD (23, 68–70) (see Supplementary Table 5 for study details). Two studies found that at baseline, decreased hippocampal volume and increased subgenual cingulate gyrus volume, respectively, were associated with better clinical response to electroconvulsive therapy (ECT) (69, 70). In addition a study of patients undergoing pharmacotherapy with fluoxetine found that greater GM volume at baseline in the ACC, insula, and right temporo-parietal cortex predicted faster and better symptom improvement (23). In keeping with this, one study of patients undergoing either pharmacotherapy with fluoxetine or CBT found that clinical remission to pharmacotherapy was predicted by greater GM volume density in the right rostral ACC, left PCC, whereas no prediction was found for CBT (68). Two studies have investigated the association between early change in GM and treatment efficacy to ECT (69, 70). These revealed early increase in hippocampal and amygdala volume (as well as in clinical symptoms) that is observable already after two ECT sessions and that this increase in volume (and the improved symptoms) predicted subsequent response to ECT (69, 70). Redlich et al. (70) also found increased GM volume in left hippocampus in patients receiving ECT over time (mean 6 weeks), pointing to a reversal of hippocampal volume loss in these patients.

#### White matter

Four studies investigated whether white matter (WM) integrity is predictive of treatment efficacy (71–74) (see Supplementary Table 5 for study details). A study of WM integrity in BD patients undergoing treatment with the atypical antipsychotic lurasidone showed that greater baseline fractional anisotropy (FA) in multiple regions, including tracts in the frontal and parietal lobes, predicted greater reduction in depressive symptoms (74). The other two studies investigated WM integrity in UD undergoing treatment with escitalopram (71, 72). One study found that remission was predicted by a pattern of higher FA in the cingulum cingulate tract (that connects the cingulate gyrus to the hippocampus) and lower FA in the stria terminalis tract (that connects the hippocampus to the hypothalamus and the rest of the limbic system) (72). The other study (71) found that lower baseline FA in the right amygdala tracts originating from the mid-brain could distinguish non-remitters from remitters. The study also showed a correlation between average FA in tracts to the right amygdala and SSRI treatment response. One final study examining WM integrity in BD undergoing chronotherapy (a combination of total sleep deprivation and morning light therapy) observed how the degree of reduction of WM integrity also biases the efficacy of treatment, so that clinical improvement negatively correlated with WM integrity (73).

#### Interim summary

Overall, findings from structural neuroimaging studies point to lower hippocampal GM volume and greater ACC volume at baseline being predictors of response to pharmacotherapy and ECT in UD.

### Non-emotional cognition to predict clinically relevant cognitive improvements

Only two studies to date have explored the impact of cognitive performance at baseline on treatment efficacy on cognition (18, 19) (see Supplementary Table 6 for study details). Both were based on a randomized controlled study of erythropoietin (EPO) treatment of patients with BD in partial remission or with treatment-resistant UD patients. The reports revealed that patients with cognitive dysfunction at baseline—as reflected by cognitive performance levels that were ≥1 SD below the norm on the targeted memory domain (18) or ≥1 on two or more domains (19) were substantially more likely to achieve treatment efficacy on cognition than those who were less impaired. This was not related to simple regression toward the mean with repeated cognitive testing since no such effect of baseline deficits was observed in the placebo group (18). In contrast, subjectively self-reported cognitive difficulties were only weakly (albeit statistically significantly) associated with better chances of achieving treatment efficacy on cognition in one (18) but not the other study (19). Taken together these two studies highlight the importance of baseline deficits in cognition for treatment efficacy on cognition. However, further studies with different interventions are necessary before any firm conclusions can be drawn regarding the impact of baseline cognition on the chances of treatment efficacy on cognitive impairment.

## Discussion

### Overall findings

**Figure 2:**
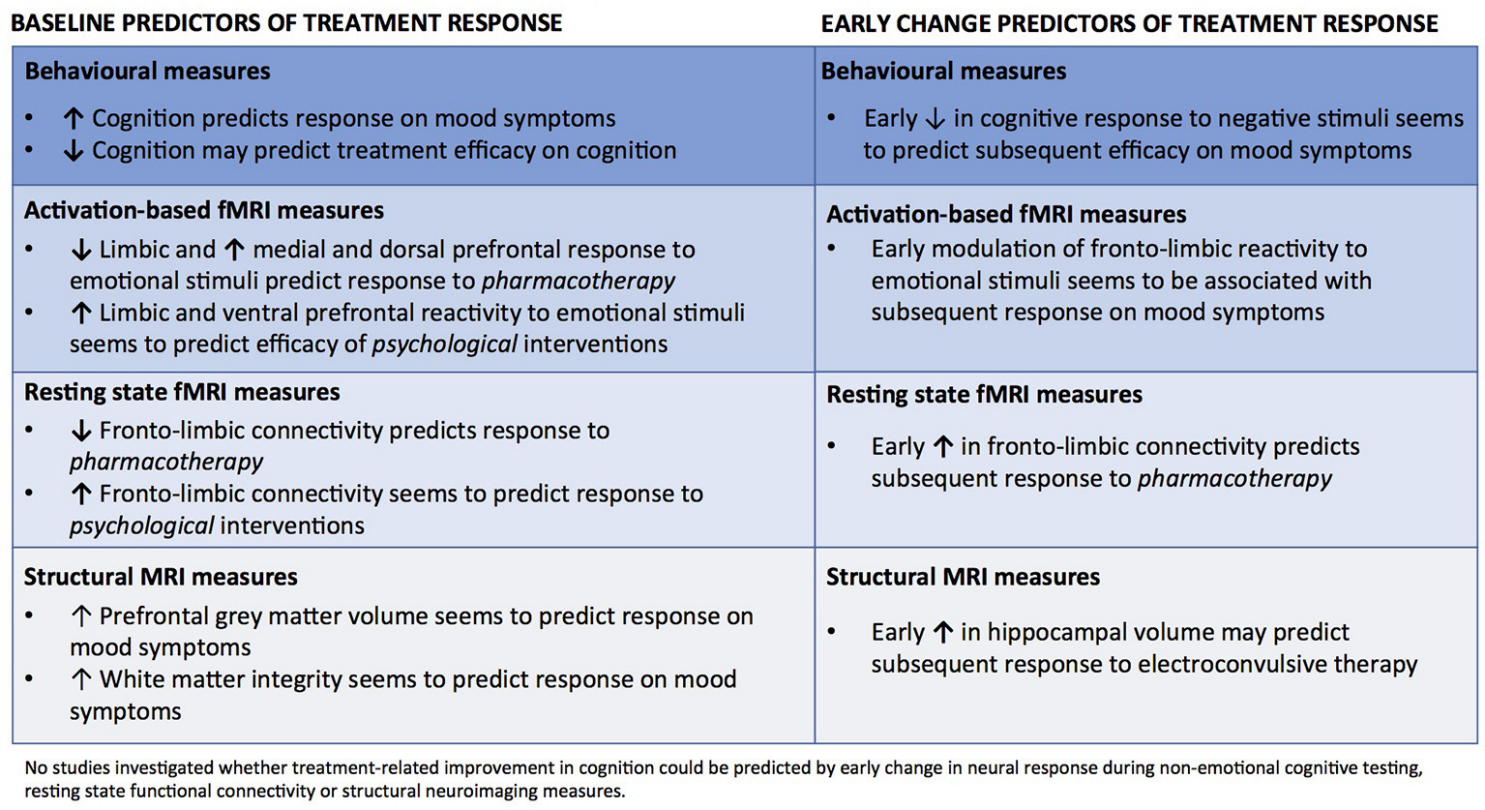
An overview of identified main predictors of treatment response.

There is a pressing need for insight into how we can most effectively adapt treatments for mood disorders to the individual patient. This systematic review identified sixty functional or structural neuroimaging and/or behavioral studies of *baseline* and *early change* biomarkers that were predictive of subsequent clinical efficacy on either depressive symptoms or cognitive impairments. An overview of the results is presented in Figure 2. The vast majority of studies (58 of 60) focused on delineating predictors of treatment response on depressive symptoms. The most consistent predictors of mood improvement were heightened PFC top-down control and greater recruitment of ACC and/or lowered limbic reactivity to negative emotional stimuli as well as high behavioral performance on non-emotional cognitive tests at baseline. Specifically, lower baseline reactivity in limbic and occipital regions coupled with greater recruitment of dorsal and medial PFC regions seems to predict better response to *pharmacotherapy*. Further, early treatment-related increase in happiness recognition and modulation of neural activity to negative stimuli in the cortico-limbic circuitry were found in several studies to predict response to several biological treatments. In contrast, *greater* baseline reactivity in limbic regions and *lower* PFC response to negative information, greater fronto-striatal connectivity as well as sustained ACC activity to reward stimuli were the most consistent predictors of response to *psychological* interventions. In addition, resting state fMRI studies showed some evidence for *lower* fronto-limbic connectivity at baseline and for an early increase in fronto-limbic connectivity predicting response to pharmacological interventions. In contrast, more positive fronto-limbic connectivity was found in resting state fMRI studies to predict response to psychotherapeutic interventions. Finally, lower baseline hippocampal GM volume, greater ACC volume and greater FA were found in structural neuroimaging studies to predict response to pharmacotherapy and ECT, while one study found that increased volume in hippocampus and amygdala after only two ECT sessions predicted later clinical response. In contrast to the large number of studies investigating predictors of mood improvement, only two studies investigated baseline predictors of treatment efficacy on cognition. These randomized, controlled studies revealed preliminary evidence for a strong association between objectively measured cognitive deficits at baseline and subsequent clinically relevant cognitive improvement. Notably, no published studies explored whether improvement in cognition could be predicted by neuronal response during cognitive testing, resting state functional connectivity or structural neuroimaging measures.

### Emotional cognition and resting state neural activity as biomarkers of efficacy on mood

Neural responses to emotional stimuli as well as functional connectivity during resting state seem to differentially predict treatment response to pharmacotherapy vs. psychological interventions and thereby aid treatment selection. For pharmacotherapy, the studies showed the best treatment response for patients exhibiting close to “normal” neural responses (i.e., low limbic reactivity and high PFC top-down control). Pharmacotherapy is hypothesized to normalize aberrant fronto-limbic activity to emotional stimuli by reducing the overwhelming influx of automatic negative cues early in treatment (75). This may explain why pharmacotherapy is more effective for patients with less aberrant neural network dysfunction. Regarding psychotherapy, the findings support the notion that heightened neural and cognitive reactivity (i.e., enhanced functional connectivity between limbic and prefrontal regions) are predictors of treatment response. It is tempting to speculate that patients with such greater limbic reactivity to emotional stimuli may also engage more emotionally in psychotherapy and thereby benefit more from this type of intervention. Further, therapy can help patients restore the network dysfunction by strengthening their conscious PFC top-down control through cognitive reappraisal and cognitive restructuring of negative automatic thoughts (42, 76). This is interesting as functional neuroimaging studies of PTSD patients treated with CBT show that excessive fear processing of emotional stimuli in the limbic system is associated with poor clinical improvement (77). This is opposite to our findings on mood disorders and therefor underlines the importance of differentiating the profiles of the patients with affective disorders including anxiety disorders.

### Non-emotional cognition, neural activity and structural measures as biomarkers of efficacy on mood

Overall, the included studies reported highly consistent evidence for better performance on non-emotional cognitive tests as a predictor of antidepressant treatment efficacy on mood symptoms. In addition, greater ACC volume and FA at baseline, as well as early volume increase in limbic regions, were predictors of response to biological treatments. Taken together, the greater cognitive performance and PFC volume in treatment responders may reflect greater capacity for neuroplasticity and is consistent with evidence for a link between good treatment prognosis and a cognitive reserve (i.e., the brain's capacity to compensate for neuropsychological damage and ability to maximize performance through recruitment of different brain networks and cognitive strategies) (8). Notably, lower hippocampal volume at baseline was also a predictor of treatment response (69, 70). This is interesting since longer duration and greater severity of depression are associated with more hippocampal volume reduction (78, 79). In light of these findings, the association between lower hippocampal volume and better treatment response could suggest that enhancement of hippocampal plasticity and neurogenesis may be key mechanisms of antidepressant drug treatment (80).

The findings from fMRI investigations on neural activity during non-emotional tests were generally unclear. Specifically, some studies found that *lower* task-related PFC activity during unsuccessful inhibition as well as a verbal working memory tasks at baseline predicted treatment response (52, 58), while other studies found that *greater* task-related PFC activity predicted treatment response (51), or that a combination of lower and higher task-related activity at baseline were predictors (i.e., lower activity in perigenual, medial OFC, and middle frontal cortices with greater activation in ventral-caudal putamen) (57). However, the overall most consistent predictor of treatment response seems to be lower response in prefrontal regions (in the absence of differences in cognitive performance) suggesting that those patients with the *most efficient* brain functioning (i.e., closer to normal with less recruitment of prefrontal resources during cognitive testing) responded better to pharmacological treatment. This adds to the notion that better brain function at baseline is a predictor of treatment response. Considering this, the discrepancies in results are likely due to the features of the different cognitive tasks employed (e.g., tests of psychomotor speed vs. executive functioning), as well as task difficulty, which have considerable effects on the strength and extent of the neural response (52). Other possible reasons for the discrepancies are patient selection (e.g., the severity of the depressive illness, illness course, inpatients vs. outpatients) and treatment modalities (different medications and clinical trial methodologies), as different pharmacological interventions might have subtle differences in mechanisms and therefore also different neuropsychological profiles for the responders.

### Non-emotional cognition as an emerging biomarker of efficacy on cognition

The emerging evidence for an association between cognitive dysfunction at baseline and patients' chances of achieving treatment efficacy on cognition has potential significance for future trials targeting cognition and for future clinical treatment of patients' cognitive deficits. Specifically, this association suggests that future intervention studies may improve their chances of demonstrating treatment efficacy by pre-screening patients for cognitive impairments before trial entry (18, 19). In addition, the use of a brief objective cognitive screener seems feasible for clinical decisions regarding which patients should be given a treatment for neurocognitive impairments rather than mood symptoms. However, notably this evidence comes from only two studies, and there is a remarkable absence of research into functional and structural neuroimaging predictors of treatment efficacy on cognition. This is a major impediment for development of better treatment strategies to target cognition. This issue and other major methodological challenges in cognition trials in mood disorders were recently addressed by a global task force of international experts in the field under the International Society for Bipolar Disorders (ISBD) (8, 16). The absence of insight into neuroimaging biomarkers for efficacy on cognition impedes insight into neurobiological targets of pro-cognitive interventions (8). Future cognition trials are therefore encouraged to implement neuroimaging assessments to increase our insight into the neurobiological predictors of cognitive improvements.

### Limitations

A limitation of this systematic review was that the included studies did not consistently report on patient demographics (e.g., the severity of the depressive illness, illness course, inpatients vs. outpatients), and hence these factors were not controlled for. Also, the inclusion criterion for study design was non-specific and did not only include randomized controlled studies, adding to a question of study quality. Adding to this, another methodological limitation to the present systematic review is that only original peer-reviewed articles were included, and a current matter in neuroimaging literature is that studies with small sample sizes or negative results may be prone to publication bias (81, 82). However, the present systematic review followed the PRISMA guidelines including multiple procedures for identification of articles, thus limiting risk of bias (21). Nevertheless this review provides a landscape overview of prediction biomarkers for treatment efficacy on depressive symptoms and cognition in both unipolar and bipolar depression by combining neuroimaging and behavioral findings. This integrated understanding of pre-treatment and early change biomarkers are only preliminary, though very promising and may have a great impact in the clinical assessments of patients to aid in efficient treatment strategies.

## Conclusion

In conclusion, this systematic review revealed several promising baseline biomarkers for prediction of treatment efficacy on mood symptoms including behavioral and neural measures of emotional and non-emotional cognition as well as cortico-limbic functional connectivity. The review also highlights a need for more studies of early treatment-related changes in these measures, since recent emerging evidence points to informative early change in emotional and non-emotional cognition before any clinical response in symptom reduction. While cognition is a new important treatment target in mood disorders, only two studies assessed the predictors of treatment response on cognition. These two reports found greater chances of treatment efficacy on cognition in patients presenting objective cognitive deficits at baseline. Nevertheless, there is a paucity of studies examining predictors of treatment-associated cognitive improvement. This highlights a pressing need for further investigation of cognitive measures and associated neuronal networks that can guide in the development of new treatment strategies targeting cognition.

## Author contributions

KM as principal investigator had the overall responsibility of the systematic review. IS conducted the literature searches, under supervision of KM. IS and HK conducted the primary and secondary screening of articles for inclusion. IS and HK wrote the initial draft of the manuscript in collaboration with KM. All authors contributed to and approved the final report.

### Conflict of interest statement

KM declares having received consultancy fees from Lundbeck. The remaining authors declare that the research was conducted in the absence of any commercial or financial relationships that could be construed as a potential conflict of interest.
