# Supplementary material for: Neural and Behavioral Predictors of Treatment Efficacy on Mood Symptoms and Cognition in Mood Disorders: A Systematic Review
Source: Front Psychiatry. 2018 Jul 26;9:337. doi: 10.3389/fpsyt.2018.00337 (PMC6071514; doi:10.3389/fpsyt.2018.00337)
Supplement: Supplementary file 1 [file Data_Sheet_1.PDF]

Supplementary material on search profile

PubMed + EMbase + PsycInfo merged

PubMed final
Systematic search, results: 995 hits

EMbase final:
Systematic search, results: 321 hits

PsycInfo final
Systematic search, results: 857 hits

Total: 995+321+857= 2173 (with duplicates)

Deletion of duplicates: 2173 – 167 (duplicates) = 2006 (no duplicates)

Final: 2006 hits

Search strings in the three databases

PubMed: Final search string: 995 hits

((((((((((("Amygdala/drug effects"[Mesh] OR "Amygdala/physiopathology"[Mesh] OR "Amygdala/therapy"[Mesh])) OR ("Hippocampus/drug effects"[Mesh] OR "Hippocampus/physiopathology"[Mesh] OR "Hippocampus/therapy"[Mesh]))) OR (((((((((("Cognition Disorders/drug therapy"[Mesh] OR "Cognition Disorders/therapy"[Mesh])) OR "Neuropsychological Tests"[Mesh]) OR "Facial Recognition"[Mesh]) OR ("Emotions/drug effects"[Mesh] OR "Emotions/drug therapy"[Mesh] OR "Emotions/therapy"[Mesh])) OR ("Facial Expression/drug effects"[Mesh] OR "Facial Expression/therapy"[Mesh])) OR ("Functional Neuroimaging/psychology"[Mesh] OR "Functional Neuroimaging/therapeutic use"[Mesh] OR "Functional Neuroimaging/therapy"[Mesh])) OR ("Magnetic Resonance Imaging/drug effects"[Mesh] OR "Magnetic Resonance Imaging/psychology"[Mesh] OR "Magnetic Resonance Imaging/therapeutic use"[Mesh] OR "Magnetic Resonance Imaging/therapy"[Mesh])) OR "Cognitive Neuroscience"[Mesh]))))) AND (((("Personality/physiology"[Mesh]) OR "Personality Inventory"[Mesh])) OR ((((("Treatment Outcome"[Mesh]) OR "Outcome Assessment (Health Care)"[Mesh]) OR "Predictive Value of Tests"[Mesh]) OR "Biomarkers, Pharmacological"[Mesh])))) AND ((("Mood Disorders"[Majr] OR "Bipolar Disorder"[Majr]) AND ("Drug Therapy"[Mesh] OR "Antidepressive Agents/therapy"[Mesh] OR "Electroconvulsive Therapy"[Mesh] OR "Treatment Outcome"[Mesh] OR "Therapeutics"[Mesh])) OR ("Mood Disorders/drug therapy"[Majr] OR "Mood Disorders/therapy"[Majr] OR "Depressive Disorder, Major/drug therapy*"[Majr] OR "Depressive Disorder, Major/therapy*"[Majr] OR "Depression/diagnosis*"[Majr])))) OR ((((((("biomarker*"[Title/Abstract] OR "marker*"[Title/Abstract] OR "biological marker"[Title/Abstract] OR “neural marker"[Title/Abstract] OR “neural markers"[Title/Abstract] OR endophenotyp*[Title/Abstract] OR surrogate*[Title/Abstract]) OR ("neuroimaging"[Title/Abstract] OR "fMRI"[Title/Abstract] OR "magnetic

resonance"[Title/Abstract] OR "cogniti*"[Title/Abstract] OR “emotional*"[Title/Abstract] OR “executive*”[Title/Abstract] OR “fronto*”[Title/Abstract] OR “limbic*”[Title/Abstract] OR “facial*”[Title/Abstract] OR “negative affective”[Title/Abstract]))) AND ("mood disorder"[Title/Abstract] OR "mood disorders*"[Title/Abstract] OR "bipolar disorder"[Title/Abstract] OR "affective disorder"[Title/Abstract] OR "unipolar disorder"[Title/Abstract] OR “major depression"[Title/Abstract] OR "unipolar depression"[Title/Abstract])) AND ("trial*"[Title/Abstract] OR “clinical response"[Title/Abstract] OR "clinical study"[Title/Abstract] OR "treatment outcome"[Title/Abstract] OR "clinical trial"[Title/Abstract] OR "RCT"[Title/Abstract] OR "randomised controlled study"[Title/Abstract] OR "randomized controlled study"[Title/Abstract] OR "treatment efficacy"[Title/Abstract] OR "treatment"[Title/Abstract]) NOT MEDLINE [SB]))))) NOT (meta[Title] OR metaanalysis[Title] OR meta-analysis[Title] OR “meta analysis"[Title] OR review[Title] OR protocol[Title] OR conference*[Title] OR tumor[Title] OR cancer*[Title] OR schizo*[Title] OR anxiety[Title] OR anxious[Title] OR adolescen*[Title] OR young[Title] OR paediatric[Title] OR pediatric[Title] OR child*[Title] OR MS[Title] OR sclerosis*[Title] OR demen*[Title] OR alzheimer*[Title] OR geno[Title] OR genotype[Title] OR genotypes[Title] OR mortality[Title] OR stroke[Title] OR prevention[Title] OR "social phobia"[Title] OR anorexia[Title] OR rat[Title] OR rats[Title] OR mice[Title] OR animal*[Title] OR CT[Title] OR CAT[Title] OR DOI[Title] OR DOT[Title] OR EROS[Title] OR PET[Title] OR MEG[Title] OR EEG[Title] OR FDG[Title] OR spect[Title] OR spectroscopy[Title] OR tomograph*[Title] OR magnetoencephalograph*[Title] OR electroencephalograph*[Title] OR "diffuse optical”[Title] OR “optical signal”[Title] OR “optical imaging”[Title] OR infrared*[Title] OR Cardiac[Title] OR “risk factor”[Title] OR “risk factors”[Title] OR Parkinsons*[Title] OR "post traumatic”[Title] OR posttraumatic[Title] OR OCD[Title] OR “compulsive disorder"[Title])

EMbase: Final search string: 321 hits

(((biomarker* or marker* or "biological marker*" or "neural marker" or "neural markers" or endophenotyp* or surrogate*).mp. [mp=title, abstract, heading word, drug trade name, original title, device manufacturer, drug manufacturer, device trade name, keyword, floating subheading word]) AND (("neuroimaging" or "fmri" or "magnetic resonance" or cogniti* or emotional* or executive* or fronto* or limbic* or facial* or "negative affective").mp. [mp=title, abstract, heading word, drug trade name, original title, device manufacturer, drug manufacturer, device trade name, keyword, floating subheading word])) AND ((("mood disorder" or "mood disorders" or "bipolar disorder" or "affective disorder" or "unipolar disorder" or "major depression" or "unipolar depression" or depression).mp. [mp=title, abstract, heading word, drug trade name, original title, device manufacturer, drug manufacturer, device trade name, keyword, floating subheading word]) AND ((trial* or "clinical response* OR clinical study OR treatment outcome OR clinical trial OR RCT OR randomised controlled study" or "randomized controlle study" or "treatment efficacy" or treat*).mp. [mp=title, abstract, heading word, drug trade name, original title, device manufacturer, drug manufacturer, device trade name, keyword, floating subheading word]))

PsycInfo: Final search string: 857 hits

((biomarker* OR marker* OR endophenotype* OR surrogate*) AND ("neuroimaging" or "fmri" or "magnetic resonance" or cogniti* or emotional* or executive* or fronto* or limbic* or facial* or "negative affective")) AND (("mood disorder" or "mood disorders" or "bipolar disorder" or "affective disorder" or "unipolar disorder" or "major depression" or "unipolar depression" or depression) AND (trial* or "clinical response*" OR "clinical study" OR "treatment outcome" OR "clinical trial" OR RCT OR "randomised controlled study" or "randomized controlled study" or "treatment efficacy" or treat*))
