# Supplementary material for: Neural and Behavioral Predictors of Treatment Efficacy on Mood Symptoms and Cognition in Mood Disorders: A Systematic Review
Source: Front Psychiatry. 2018 Jul 26;9:337. doi: 10.3389/fpsyt.2018.00337 (PMC6071514; doi:10.3389/fpsyt.2018.00337)
Supplement: Supplementary file 2 [file Table_1.pdf]

**Table 1: Emotional cognition - Biomarkers of treatment response on mood symptoms**

| Author | Paradigm | Measure | Patients | N | Study design | Treatment group | Finding |
|---|---|---|---|---|---|---|---|
| Carl et al. (2016) | Monetary incentive delay task (MID) | fMRI and behaviour | MDD | 33 TGs, 20 HCs | Non-RCT (only HC group) | BATD (16 sessions) | At baseline, responders showed greater sustained activation in the ACC during reward outcomes compared to non-responders. Also, greater change in reaction times during reward trials (i.e. faster response at run 2) at baseline predicted treatment response. |
| Chen et al. (2007) | Facial emotion processing paradigm | fMRI | MDD | 17 TGs | Open-label uncontrolled | SSRI fluoxetine (8 weeks) | At baseline, responders showed increased ACC activation to fearful and angry faces. |
| Costafreda et al. (2009b) | Facial emotion processing paradigm | fMRI | UD | 16 TGs | Open-label uncontrolled | CBT (16 sessions) | At baseline, the functional connectivity during processing of sad faces at the lowest and highest intensities identified patients, who had a full clinical response to CBT. Regions that showed the greatest contribution to the prediction of clinical remission included the ACC, superior and middle frontal cortices, paracentral cortex, superior parietal cortex, precuneus and cerebellum. |

| | | | | | | | |
|---|---|---|---|---|---|---|---|
| Davidson et al. (2003) | Emotional processing to emotional laden pictures | fMRI | MDD | 12 TGs, 5 HCs | Non-RCT (only HC group) | SNRI venlafaxine (8 weeks) | At baseline, responders showed greater relative ACC activation in response to the negative pictures compared to HC. Also, at baseline responders showed increased acitvity in posterior cingulate gyrus and precuneus in response to positive pictures compared to non-responders. |
| Delaveau et al. (2016) | Emotional sel-referential processing to emotional laden IAPS and EPS pictures | fMRI | UD | 13 TGs, 12 PGs, 14 HCs | RCT double blind, placebo-controlled | Atypical antidepressant (melatonin and serotonin receptor antagonist) agomelatine (24 weeks) | At baseline, remitters showed lower activation in the DMPFC10 (rostral part), PCC and DLPFC during self-referential processing compared to non-remitters. |
| Fu et al. (2008) | Facial emotion processing paradigm | fMRI | MDD | 16 TGs, 16 HCs | Non-RCT (only HC group) | CBT (16 sessions) | At baseline, responders showed decreased DACC activity to sad faces compared to non-responders, but closer to HC activity levels. |

| | | | | | | | |
|---|---|---|---|---|---|---|---|
| Furey et al. (2013) | Facial emotion processing paradigm | fMRI | MDD | 15 TGs, 21 HCs | RCT double blind, placebo-controlled | Anticholinergic antidepressant scopalomin (7x3 infusions) | At baseline, responders showed decreased bilateral middle occipital cortex activity, selectively during the stimulus-processing components of the emotion working memory task (no correlation during the identity task). Following short-term scopalomine administration, responders exhibited increased activity in bilateral middle occipital cortex (relative to baseline activity) during encoding and recognition of faces with (task-irrelevant) emotional expressions. |
| Godlewska et al. (2016) | Facial emotion processing paradigm | fMRI | MDD | 35 TGs, 29 HCs | Non-RCT (only HC group) | SSRI escitalopram (6 weeks) | At baseline, patients showed heightened insula and DACC reactivity to fear vs. happy faces compared to HC, but this did not significantly predict treatment response. Instead, at Week 1, future responders showed a greater decrease in ACC, insula, Amy and thalamus reactivity to fearful vs. happy faces. |

| | | | | | | | |
|---|---|---|---|---|---|---|---|
| Light et al. (2011) | Positive emotion regulation paradigm with IAPS pictures | fMRI | MDD | 19, TGs, 19 HCs | Non-RCT (only HC group) | SNRI venlafaxine (N=9) or SSRI fluoxetine (N=10) (8 weeks) | At baseline, responders showed decreased RVLPFC activity during attempts to dampen their experience of positive emotion in response to positive visual stimuli. |
| Lisiecka et al. (2011) | Facial emotion processing paradigm | fMRI | MDD | 23 TGs, 27 HCs | RCT open-label | SNRI venlafaxine (N=13) or NaSSA mirtazapine (N=10) | At baseline, responders had higher functional OFC connectivity in the left precentral gyrus and internally within the right middle OFC. |
| Miller et al. (2013) | Emotional sel-referential processing to emotional laden words | fMRI | MDD | 17 TGs | Open-label uncontrolled | SSRI escitalopram (8 weeks) | At baseline, responders showed decreased responses to negative self-referential words in midbrain, DLPFC, paracingulate, ACC, thalamus and caudate nuclei. |
| Redlich et al. (2017) | Facial emotion processing paradigm | fMRI | MDD | 19 TGs, 20 PGs, 19 HCs | Non-randomized controlled trial (both CG and HC group) | ECT (9-12 sessions) | Both treatment groups showed increased Amy reactivity to sad faces at baseline compared to HC. No significant finding on predictors of treatment response. |

| | | | | | | | |
|---|---|---|---|---|---|---|---|
| Ritchey et al. (2011) | Emotional processing to emotional laden IAPS pictures | fMRI | MDD | 15 TGs, 7 HCs | Non-RCT (only HC group) | CBT (20 sessions) | At baseline, responders to CBT showed heightened DLPFC and ATL activity in response to negative pictures and pictures in general. |
| Rizvi et al. (2013) | Emotional processing to emotional laden IAPS pictures | fMRI | MDD | 21 TGs, 11 HCs | Non-RCT (only HC group) | SSRI fluoxetine and antipsychotic olanzapine (6 weeks) | At baseline, responders had greater premotor and posterior cingulate cortex activity while viewing negative images compared to non-responders and HC. |
| Ruhé et al. (2012) | Facial emotion processing paradigm | fMRI | MDD | 20 TGs, 20 HCs | Non-RCT (only HC group) | SERT paroxetine (12 weeks) | At baseline, responders showed relatively lower bilateral Amy reactivity in response to negative facial expressions compared to non-responders, which normalized towards that of healthy controls after short-term paroxetine administration. Also, at week 6 responders showed increased activity in lower dorsal regions (DLPFC and DMPFC) to negative faces (towards ‘normal ‘levels) relative to non-responders. |
| Samson et al. (2011) | Emotional processing to emotional laden faces | fMRI | MDD | 21 TGs, 12 HCs | Non-RCT (only HC group) | NaSSA mirtazapine or SNRI venlafaxine (4 weeks) | At baseline, responders showed heightened DMPFC and PCC reactivity to negative emotional faces. |

| | | | | | | | |
|---|---|---|---|---|---|---|---|
| Siegle et al. (2006) | Emotional sel-referential processing to emotional laden words | fMRI | MDD | 14 TGs, 21 HCs | Non-RCT (only HC group) | CBT (16 sessions) | At baseline, responders to CBT showed sustained reactivity to emotional stimuli with decreaed SCC reactivity and increased amygdala reactivity. |
| Siegle et al. (2012) | Emotional sel-referential processing to emotional laden words | fMRI | MDD | 49 TGs, 35 HCs | Non-RCT (only HC group) | CBT (16-20 sessions) | At baseline, responders showed the lowest sustained sgACC reactivity in response to negative words. |
| Strakowski et al. (2016) | Continuous Performance Task with Emotional and Neutral Distracters/pictures (CPT-END) from IAPS: visual odd ball paradigm | fMRI | BD I | 42 TGs, 41 HCs | Pseudo-RCT open-label (two-armed TG and HC) | Moodstabiliser lithium (N=19) or antipsychotic quetiapine (N=23) (8 weeks) | After short-term treatment (1 week), future patients who achieved remission exhibited increased activation in temporal, medial PFC and posterior accessory cortical areas when processing emotional stimuli. |
| Szczepanik et al. (2016) | Facial emotion processing paradigm | fMRI | MDD | 14 TGs/PGs, 15 HCs | RCT double blind, placebo-controlled | Anticholinergic antidepressant scopalomin (7x3 infusions) | At baseline, decreased Amy reactivity during explicit processing (selective attention) of sad faces predicted treatment responders. The observed effect was uniqely associated with attentional focus on sad faces (explicit vs. implicit), and also processing of sad faces without competing meaningful stimuli was not significantly |

| | | | | | | | |
|---|---|---|---|---|---|---|---|
| | | | | | | | related to treatment response. |
| Tranter et al. (2009) | Facial emotion processing paradigm | Behaviour | UD | 69 TGs, 108 HCs | RCT open-label | SSRI citalopram (N=39) or NRI reboxetine (N=30)) (6 weeks) | After short term treatment (2 weeks), there was a significant positive correlation between increased accuracy in recognition of happy faces and later clinical improvement. |
| Vai et al. (2015) | Facial emotion processing paradigm | fMRI | BD I | 37 TGs, 35 HCs | Non-RCT (only HC group) | Chronotherapeutics: repeated total sleep deprivation combined with light therapy (6 days) | At baseline, non-responders showed decreased DLPFC, ACC and insula activity to sad faces compared to responders. Increased ACC and MPFC activity to sad faces was associated with successfull antidepressant treatment. In respect to baseline, responders showed after treatment a significantly increased top-down connectivity from DLPFC to ACC and a significantly reduced modulatory effect of the task on the connectivity from Amy to DLPFC, while non-responders did not show any change. A successful antidepressant treatment was associated with an increased functional activity and |

| | | | | | | | |
|---|---|---|---|---|---|---|---|
| | | | | | | | connectivity within cortico-limbic networks during the implicit regulation of affective states. |
| Vai et al. (2016) | Facial emotion processing paradigm | fMRI | MDD | 33 TGs, 31 HCs | Non-RCT (only HC group) | SSRI (6 weeks) | At baseline, non-responders showed a reduced endogenous connection from Amy to PFC compared to HC when processing of fearful emotional faces. These connections were inhibitory in non-responders, whereas HC and responders showed excitatory bottom-up connections. Also, at baseline non-responders had an increased and excitatory modulatory effect from ACC to Amy compared to HC, who instead presented inhibitory top-down control from ACC to Amy. Responders and HC did not significantly differ among themselves. |
| Victor et al. (2013) | Facial emotion processing paradigm | fMRI | MDD | 10 TGs, 10 HCs | Non-RCT (only HC group) | SSRI sertralin (8 weeks) | At baseline, pgACC responses to SN-HN correlated positively with clinical improvement during treatment. |

| | | | | | | | |
|---|---|---|---|---|---|---|---|
| Walsh et al. (2017) | Monetary incentive delay task (MID) | fMRI | MDD | 33 TGs, 20 HCs | Non-RCT (only HC group) | BATD (16 sessions) | At baseline, responders showed greater connectivity between the left putamen and paracingulate gyrus during reward anticipation. In addition, MDD participants with greater attenuation of connectivity between several frontostriatal seeds, and midline subcallosal cortex and left paracingulate gyrus demonstrated improved response to BATD. |
| Williams et al. (2015) | Facial emotion processing paradigm | fMRI | MDD | 80 TGs, 34 HCs | RCT open-label | SSRI escitalopram or SSRI sertralin or SNRI venlafaxine (8 weeks) | At baseline, responders to either SSRI or SNRI showed decreased Amy reactivity to emotions signalling reward and threat, while specifically non-response to SNRI was predicted by increased Amy reactivity to sad emotion. |
