# Supplementary material for: Neural and Behavioral Predictors of Treatment Efficacy on Mood Symptoms and Cognition in Mood Disorders: A Systematic Review
Source: Front Psychiatry. 2018 Jul 26;9:337. doi: 10.3389/fpsyt.2018.00337 (PMC6071514; doi:10.3389/fpsyt.2018.00337)
Supplement: Supplementary file 3 [file Table_2.pdf]

| Table 2: Non-emotional cognition - Biomarkers of treatment response on mood symptoms | | | | | | | |
|---|---|---|---|---|---|---|---|
| **Author** | **Paradigm** | **Measure** | **Patients** | **N** | **Study design** | **Treatment group** | **Finding** |
| Cléry-Melin et al. (2017) | The domains and their composite tests were as follows: Processing speed, attentive flexibility, planning, and task shifting (TMT A-B); attention (d2 neurocognitive drawing test) | Behaviour | MDD | 508 TGs | Open-label uncontrolled | Atypical antidepressant (melatonin and serotonin receptor antagonist) agomelatine (8 weeks) | At baseline, responders showed higher attention performance. |
| Crane et al. (2017) | Parametric Go/No-go task | fMRI and behaviour | MDD | 29 TGs | Open-label uncontrolled | SSRI escitalopram or SNRI duloxetine (10 weeks) | At baseline, responders showed more unsuccessful inhibition (i.e., more performance errors) as well as less recruitment (i.e. lower activation) of brain areas important for cognitive control and/or interference resolution, including the ACC, left VMPFC and right VLPFC |

| | | | | | | | |
|---|---|---|---|---|---|---|---|
| Dunkin et al. (2000) | The domains and their composite tests were as follows: Basic Attention (WAIS-R Digit Span); Information Processing Speed: (TMT-A, STWC, WAIS-R Digit Symbol); Language (Boston Naming Test); Visuoperceptual Abilities (WAIS-R Block Design, Rey-Osterreith Complex Figure Copy, Benton Facial Recognition Test); Verbal Memory (RAVLT); Nonverbal Memory (WMS-R Visual Reproduction, Rey-Osterreith Complex Figure-3-min delayed recall); Executive Functions (WCST, Auditory Consonant Trigrams (ACT), COWAT, Stroop Task-Part C, and TMT-B) | Behaviour | MDD | 22 TGs | RCT double blind, placebo-controlled | SSRI fluoxetine (8 weeks) | At baseline, responders showed higher performance on measures of executive functioning compared to non-responders. |
| Furtado et al. (2012) | WTAR, BVMT, RAVLT, TMT A-B, WAIS III Digit-Span subtest, COWAT | fMRI and behaviour | MDD | 42 TGs, 46 HCs | Non-RCT (only HC group) | rTMS (6 weeks) | There was no significant difference in baseline neurocognitive profiles and medial temporal lobe (MTL) volumes between eventual treatment responders and non-responders. |

| | | | | | | | |
|---|---|---|---|---|---|---|---|
| Gudayol-Ferre et al. (2010) | The domains and their composite tests were as follows: Premorbid intellectual functioning (WAIS III vocabulary subtest); Working memory (WAIS III Digit span, SWM); Verbal learning and memory (RAVLT); Visual learning and memory (PAL, DMS); Mental and motor processing speed (STWC); Attention (RVP, WAIS III Digit span); Executive function, response inhibition (Stroop test colors); Executive function, fluency (COWAT); Executive functions; attentional set-shifting (ID/ED); Executive function, planning (SOC) | Behaviour | MDD | 64 TGs | Open-label uncontrolled | SSRI fluoxetine (4 weeks) | At baseline, responders showed a combination of a good performance in variables of attention and low performance in planning. |
| Hernández-Ribas et al. (2013) | Word-generation task (based on COWAT) | fMRI and behaviour | MDD | 21 TGs/PGs, 15 HCs | RCT double blind, placebo-controlled | rTMS (15 sessions) | At baseline, responders displayed a combination of lower activity in perigenual, medial OFC and middle frontal cortices, and greater activation in the ventral-caudal putamen during a word-generation task compared to non-responders |

| | | | | | | | |
|---|---|---|---|---|---|---|---|
| Herrere-Guzman et al. (2008) | The domains and their composite tests were as follows: Premorbid intellectual functioning: (WAIS III vocabulary subtest); Short term and Working memory (WAIS III Digit span, DMS); Verbal learning and memory (RAVLT); Visual learning and memory (PRM, PAL, Spatial Recognition Memory); Mental and motor processing speed (MTS Visual Search, Reaction Time); Sustained Attention (RVP); Executive function, Response inhibition (STWC); Executive function, Verbal fluency (COWAT); Executive function, Attention shifting (ID/ED); Executive function, Strategy (SWM); Executive functions, Planning (SOC). | Behaviour | MDD | 20 TGs | Open-label uncontrolled | Atypical antidepressant (norepinephrine–dopamine reuptake inhibitor) bupropion (8 weeks) | At baseline, responders showed deficits in visual memory and mental processing speed. |

| | | | | | | | |
|---|---|---|---|---|---|---|---|
| Hoy et al. (2012) | Digit Span Forward & Backward, Hopkins Verbal Learning Test (HVLT), Brief Visuospatial Memory Test (BVMT), Controlled Oral Word Association Test (COWAT) | Behaviour | BD & MDD | 137 TGs | Open-label uncontrol led | rTMS (4-6 weeks) | Initial improvements in performance on immediate visuospatial memory were significantly related to eventual treatment response, with visuospatial improvement being a significant predictor of degree of eventual improvement in a near significant regression model. |
| Kampf-Sherf et al. (2004) | TMT A-B, Purdue Pegboard, Rey–Osterrieth Complex Figure Test, RAVLT, BVRT, WAIS–R Arithmetic+Block Design+Similarities subtests, Hooper Visual Organization Test, Visual Frequency of Occurrence Test. | Behaviour | MDD | 55 TGs | Open-label uncontrol led | SSRI (6 weeks) | At baseline, responders were characterized by better functioning in ‘‘simple’’ tasks (simple and fast responses, and/or little information processing is required) and by worse functioning in ‘‘complex’’ tasks (cognitive tasks that require patients to wait and/or to process large amounts of information) compared to nonresponders. |

| | | | | | | | |
|---|---|---|---|---|---|---|---|
| Langenecker et al. (2007) | Parametric Go/No-go task | fMRI and behaviour | MDD | 15 TGs, 22 HCs | Non-RCT (only HC group) | SSRI citalopram (10 weeks) | At baseline, responders showed increased rostral ACC activation during incorrect inhibitory trials (commissions). |
| Raes et al. (2008) | The domains and their composite tests were as follows: Memory specificity (Autobiographical memory test); Executive control (Letter-Number Sequencing test). | Behaviour | BD & UD | 25 TGs | Open-label uncontrolled | ECT (9-12 sessions) | The greater the proportion of OGMs retrieved before treatment, the greater the increase (or the smaller the decrease) in depression scores after treatment stops. |
| Taylor et al. (2006) | The domains and their composite tests were as follows: psychomotor speed (COWAT, STWC, Wisconsin card sorting task, WAIS III Digit symbol & Block design & Digit Span & Vocabulary) | Behaviour | UD | 37 TGs | RCT open-label | SSRI fluoxetine (12 weeks) | At baseline, responders displayed better mental processing speed, significantly predicted by better performance on verbal fluency (using COWAT FAS). |
| Walsh et al. (2007) | N-back verbal working memory | fMRI and behaviour | MDD | 20 TGs | Open-label uncontrolled | SSRI fluoxetine (8 weeks) | At baseline, responders showed lower linear load-response (decreased activity) during a verbal working memory task in the DACC. |
