# Supplementary material for: Neural and Behavioral Predictors of Treatment Efficacy on Mood Symptoms and Cognition in Mood Disorders: A Systematic Review
Source: Front Psychiatry. 2018 Jul 26;9:337. doi: 10.3389/fpsyt.2018.00337 (PMC6071514; doi:10.3389/fpsyt.2018.00337)
Supplement: Supplementary file 4 [file Table_3.pdf]

Table 3: Combined emotional and non-emotional cognition - Biomarkers of treatment response on mood symptoms

Author	Paradigm	Measure	Patients	N	Study design	Treatment group	Finding
Etkin et al. (2015)	Not otherwise specified. Participants completed a computerized battery of tests designed to evaluate a range of cognitive and emotional capacities including attention, working memory, psychomotor response speed, cognitive flexibility of task shifting, response inhibition, verbal memory, processing speed, decision speed, emotion identification, and emotional biasing of memory for faces	Behaviour	MDD	665 TGs, 336 HCs	RCT double-blind	SSRI escitalopram or SSRI sertraline or SNRI venlafaxine (8 weeks)	A subgroup of depressed participants (approximately one-quarter of patients) were found to be impaired across most cognitive tests relative to the healthy norm, from which they could be discriminated with 91% accuracy. These patients with generally impaired cognitive task performance had poorer treatment outcomes. For this impaired subgroup, task performance furthermore predicted remission on the QIDS-SR16 at 72% accuracy specifically following treatment with escitalopram but not the other medications.

Gordon et al. (2015)	The domains and their composite tests were as follows: Information processing speed (switching of attention task); Cognitive flexibility (verbal interference); Attention (continuous performance test); Response inhibition (Go/no-go task); Working memory (Digit span); Motor coordination (Motor tapping); Executive function (Maze); Emotion identification accuracy & Speed of emotion identification (Emotion identification)	Behaviour	MDD	467 TGs, 336 HCs	RCT open-label	SSRI escitalopram (N=157) or SSRI sertraline (N=175) or SNRI venlafaxine (N=135) (8 weeks)	The cognitive assessment battery (including 8 general cognition tasks and 1 emotional cognition task) was able to identify 32%–62% of individual participants who will not remit after 8 weeks of treatment with an actionable level of certainty (80%).
