# Supplementary material for: Neural and Behavioral Predictors of Treatment Efficacy on Mood Symptoms and Cognition in Mood Disorders: A Systematic Review
Source: Front Psychiatry. 2018 Jul 26;9:337. doi: 10.3389/fpsyt.2018.00337 (PMC6071514; doi:10.3389/fpsyt.2018.00337)
Supplement: Supplementary file 5 [file Table_4.pdf]

Table 4: Resting state functional connectivity - Biomarkers of treatment response on mood symptoms

Author	Paradigm	Measure	Patients	N	Study design	Treatment group	Finding
Cano et al. (2016)	Functional network connectivity	rs-fMRI	MDD	13 TGs, 10 HCs	Non-RCT (only HC group)	ECT (9 sessions)	An early significant intralimbic FC decrease significantly predicted a later increase in limbic–prefrontal FC, which in turn significantly predicted clinical improvement at the end of an ECT course.
Cheng et al. (2017)	Functional 'fractional amplitude of low-frequency fluctuation' (fALFF)	rs-fMRI	MDD	38 TGs, 74 HCs	Non-RCT (only HC group)	SSRI escitalopram (8 weeks)	After 5 h the antidepressant induced a significant decrease in the signal in the occipital cortex and an increase in the DLPFC and DMPFC and middle cingulate cortex, which predicted later clinical response.
Crowther et al. (2015)	Functional network connectivity	rs-fMRI	MDD	23 TGs, 20 HCs	Non-RCT (only HC group)	BATD (20 sessions)	At baseline, responders showed greater connectivity of the right insula with the right middle temporal gyrus.
Downar et al. (2014)	Functional network connectivity	rs-fMRI	UD & BD	47 TGs	Open-label uncontrolled	rTMS (20 sessions)	At baseline, responders showed increased connectivity within reward pathways, including left VMPFC, the ventral tegmental area, and striatum compared with non-responders

Dunlop et al. (2017)	Functional network connectivity	rs-fMRI	MDD	122 TGs	RCT double blind, placebo-controlled	SSRI (escitalopram, duloxetine) or CBT (12 weeks)	Positive connectivity within the SCC and (1) the left VLPFC/insula; (2) left VMPFC; and (3) dorsal midbrain was associated with remission with CBT, whereas negative connectivity in these areas was associated with better response to medication.
Fang et al. (2017)	Functional network connectivity	rs-fMRI	MDD	17 TGs, 21 PGs	NOS	tVNS (4 weeks)	The insula activation level during the first stimulation session in the tVNS group was significantly associated with the clinical improvement at the end of the four-week treatment.
Ge et al. (2017)	Functional network connectivity	rs-fMRI	MDD	18 TGs, 21 HCs	Non-RCT (only HC group)	rTMS (6 weeks)	At baseline, responders showed higher functional connectivity in the ACC/VMPFC within the anterior default mode network as well as in the DACC/insula within the salience network
Liston et al. (2014)	Functional network connectivity	rs-fMRI	MDD	17 TGs, 35 HCs	Non-RCT (only HC group)	TMS (5 weeks)	Prior to treatment, functional connectivity in depressed patients was abnormally elevated within the DMN and diminished within the CEN, and connectivity between these two networks was altered. Baseline subgenual connectivity predicted subsequent clinical improvement.

Salomons et al. (2014)	Functional network connectivity	rs-fMRI	UD & BD	25 TGs	Open-label uncontrolled	rTMS (4 weeks)	At baseline, responders showed higher cortico-cortical connectivity (dmPFC-subgenual cingulate and subgenual cingulate to dorsolateral PFC) as well as lower cortico-thalamic, cortico-striatal, and cortico-limbic connectivity.
van Waarde et al. (2015)	Functional network connectivity	sMRI, rs-fMRI	MDD	45 TGs	Open-label uncontrolled	ECT (19 sessions)	The study revealed two resting-state networks with significant classification accuracy after correction for multiple comparisons. A network centered in the dorsomedial prefrontal cortex (including the dorsolateral prefrontal cortex, orbitofrontal cortex and posterior cingulate cortex) showed a sensitivity of 84% and specificity of 85%. Another network centered in the anterior cingulate cortex (including the dorsolateral prefrontal cortex, sensorimotor cortex, parahippocampal gyrus and midbrain) showed a sensitivity of 80% and a specificity of 75%.
