# Supplementary material for: Neural and Behavioral Predictors of Treatment Efficacy on Mood Symptoms and Cognition in Mood Disorders: A Systematic Review
Source: Front Psychiatry. 2018 Jul 26;9:337. doi: 10.3389/fpsyt.2018.00337 (PMC6071514; doi:10.3389/fpsyt.2018.00337)
Supplement: Supplementary file 6 [file Table_5.pdf]

Table 5: Structural abnormalities - Biomarkers of treatment response on mood symptoms

Author	Paradigm	Measure	Patients	N	Study design	Treatment group	Finding
Bollettini et al. (2015)	White-matter (using FA)	DTI-MRI	BD	70 TGs	Open-label uncontrolled	Chronotherapy (a combination of total sleep deprivation and morning light therapy) (3 cycles, 1 week)	Increased mean and radial water diffusivity correlated with poor antidepressant response to TSD+LT in core WM tracts: including corpus callosum, corona radiata, cingulum bundle, superior longitudinal fasciculus, inferior fronto-occipital fasciculus, and thalamic radiation.
Chen et al. (2007)	Grey matter volume (using VBM)	sMRI	MDD	17 TGs	Open-label uncontrolled	SSRI fluoxetine (8 weeks)	At baseline, responders showed greater GM volume in ACC, insula, and right temporo-parietal cortex.
Costafreda et al. (2009a)	Grey matter volume (using VBM)	sMRI	MDD	30 TGs	Open-label uncontrolled	SSRI fluoxetine (8 weeks) or CBT (16 weeks)	At baseline, responders to SSRI showed greater grey matter density in the right rostral anterior cingulate cortex, left posterior cingulate cortex, left middle frontal gyrus, and right occipital cortex. Regions which predicted residual symptoms were the orbitofrontal cortices bilaterally, right superior frontal cortex and left hippocampus. The structural neuroanatomy did not show a significant prediction of clinical remission to CBT.

DeLorenzo et al. (2013)	White-matter (using FA)	DW-MRI	MDD	18 TGs	Open-label uncontrolled	SSRI escitalopram (8 weeks)	Results indicate that average FA in DW-MRI-derived tracts to the right Amy was significantly lower in non-remitters than remitters at baseline. In addition, regarding connectivity to Amygdala there was a significant correlation between average FA in tracts to the right amygdala and SSRI treatment response, and this was not found to be significant for connectivity to hippocampal.
Joshi et al. (2017)	Grey matter volume (using VBM)	sMRI	MDD and BD	29 TGs, 32 HCs	Non-RCT (only HC group)	ECT (3 sessions)	At baseline, decreased hippocampal volume predicted volume increases and subsequent clinical response. Results further indicate that both clinical response to ECT and ECT-induced changes in volume occur rapidly. That is, significant changes in both clinical scores and hippocampal and amygdala volume are observable after the 2nd ECT session.

Korgaonkar et al. (2014)	White-matter (using FA)	DTI-MRI	MDD	74 TGs, 34 HGs	RCT double blind, placebo-controlled	SSRI escitalopram or SSRI sertraline or SNRI venlafaxine (8 weeks)	A cross-validated logistic regression model demonstrated that altered connectivity for the cingulum part of the cingulate and stria terminalis tracts significantly predicted remission independent of demographic and clinical measures with 62% accuracy. Prediction improved to 74% when age was added to this model.
Lan et al. (2017)	White-matter (using FA)	DTI-MRI	BD I (N=2); BDI II (N=11); NOS (N=5)	18 TGs	Open-label uncontrolled	Atypical antipsychotic (lurasidone) (8 weeks)	Greater FA predicted antidepressant treatment response in multiple regions of the mean FA skeleton bilaterally, including tracts in the frontal and parietal lobes, but notably did not include the cerebellum.
Redlich et al. (2016)	Grey matter volume (using VBM)	MRI	MDD	23 TGs, 23 PGs, 21 HCs	Non-randomized controlled trial (both CG and HC group)	ECT (9-12 sessions)	The study results indicated a positive association between baseline subgenual cingulate volume and individual ECT response. A relatively small degree of structural impairment in the subgenual cingulate cortex before therapy seems to be associated with successful treatment with ECT.
