# Supplementary material for: Neural and Behavioral Predictors of Treatment Efficacy on Mood Symptoms and Cognition in Mood Disorders: A Systematic Review
Source: Front Psychiatry. 2018 Jul 26;9:337. doi: 10.3389/fpsyt.2018.00337 (PMC6071514; doi:10.3389/fpsyt.2018.00337)
Supplement: Supplementary file 7 [file Table_6.pdf]

Table 6: Cognitive performance - Biomarkers of treatment response on cognition

Author	Paradigm	Measure	Patients	N	Study design	Treatment group	Finding
Miskowiak et al. (2016)	RAVLT (objective) and CPFQ (subjective)	Behaviour	BD & UD	39 TGs, 40 HCs	RCT double-blind	EPO (8 weeks / 8 infusions)	For EPO-treated patients with objective memory dysfunction at baseline (n = 16) (defined as RAVLT total recall ≤ 43), the odds of a clinically relevant memory improvement were increased by a factor of 290.6 (95% CI, 2.7-31,316.4; P = .02) compared to patients with no baseline impairment (n = 23). Subjective cognitive complaints (measured with the Cognitive and Physical Functioning Questionnaire) and longer illness duration were associated with small increases in patients' chances of treatment efficacy on memory (53% and 16% increase, respectively; P ≤ .04).

Ott et al. (2016)	Objective cognitive function covering domains of attention, memory and executive function (RAVLT, RBANS coding, verbal fluency test with the letter D, WAIS-III letter-number sequencing, TMTB, RVP); subjective cognitive complaints (CPFQ)	Behaviour	BD & UD	39 TGs, 40 HCs	RCT double-blind	EPO (8 weeks / 8 infusions)	EPO improved speed of complex cognitive processing across affective disorders at weeks 9 and 14. In EPO-treated patients, baseline cognitive impairment increased the odds of treatment-efficacy on cognition at weeks 9 and 14 by a factor 9.7 and 9.9 respectively. Subjective cognitive complaints did not affect chances of treatment-efficacy.
